# A comprehensive interventional program based on the needs and concerns related to female genital cosmetic surgeries: protocol for a multistage mixed methods study

**DOI:** 10.1186/s12978-023-01717-6

**Published:** 2023-12-04

**Authors:** Zahra Ghorbani, Zahra Behboodi Moghadam, Mojgan Mirghafourvand, Faezeh Vahidnia, Elham Ebrahimi

**Affiliations:** 1 Department of Midwifery and Reproductive Health, School of Nursing and Midwifery, Tehran University of Medical Sciences, Tehran, Iran; 2 Department of Midwifery, Bonab Branch, Islamic Azad University, Bonab, Iran; 3 Social Determinants of Health Research Center, Tabriz University of Medical Sciences, Tabriz, Iran; 4 Medical Philosophy and History Research Center, Tabriz University of Medical Sciences, Tabriz, Iran

**Keywords:** Genital cosmetic surgery, Empowerment, Sexual health, Mix-method, Qualitative

## Abstract

**Background:**

Dissatisfaction with one's body and the subsequent rise in the cosmetic surgery trend pose significant public health concerns today. One unusual cosmetic surgical procedure involves enhancing the genital area. Numerous healthy women and girls have recently sought Genital Cosmetic Surgery (GCS) for beauty or improved sexual performance. There is a concern that this phenomenon may be linked to developing a new standard for vulvovaginal appearance. This stringent standard could potentially adversely affect women's mental health in the future, growing feelings of insecurity and possibly leading teenagers to consider plastic surgery.

Implementing empowering and awareness-raising programs for women and girls is crucial, especially in light of the constantly evolving gender norms and the medicalization of sexuality and beauty as social constructs. It is essential that such training is integrated into comprehensive sexual education programs for adolescents. These efforts align perfectly with the SDG, recognizing that education in sexual and reproductive health, ensuring access to health, and empowering women are fundamental rights for women and girls. To accomplish these objectives, we will conduct this study to elucidate the needs and concerns related to the increasing trend of GCS. By doing so, we can concentrate on the factors motivating women to undergo GCS. This approach will enable us to develop effective interventions to empower women and girls considering GCS, thus enhancing their sexual and reproductive health.

**Methods and objectives:**

The objectives of this multistage exploratory sequential mixed-method study will be structured into three phases:

First phase: qualitative study.

1. In-depth interviews will be conducted to elucidate the needs and concerns associated with GCS with women with a history of GCS, spouses of willing participants, and women actively seeking these procedures.
2. A literature review in parallel with the qualitative phase will be conducted to gain insights into the needs and concerns of women worldwide considering GCS.

Second phase: program design.

1. To formulate an intervention grounded in the primary priorities identified during the qualitative stage and informed by the literature review.
2. To prioritize the needs and concerns of women seeking GCS and to validate and endorse the intervention through input from an expert panel.

Third phase: quantitative study.

To assess and determine the effectiveness of the intervention designed to address the needs and concerns of women applying for GCS procedures.

**Discussion:**

This study marks the first attempt to design and assess an intervention addressing the needs and concerns of cosmetic surgeries performed on the female genital and reproductive system. The hope is that this study's compilation and implementation will yield substantial evidence and documentation regarding the impact of educational interventions on women's and girls' sexual and reproductive empowerment. Given the rising prevalence of GCS among unmarried teenagers, this approach is of utmost significance. It underscores the necessity for gynecological and midwifery service providers to have comprehensive guidance on GCS. Such guidance can be an essential resource for healthcare providers in this field.

## Plain language summary

Contemporary concerns about body dissatisfaction and the growing trend of cosmetic surgery, including unusual trends of genital cosmetic surgery (GCS), are raising public health issues. This study addresses the needs and concerns related to the increasing trend of GCS among women.

A multistage exploratory sequential mixed-method study will be structured into three phases, incorporating both quantitative and qualitative components:

1. A qualitative phase involving in-depth interviews and a literature review
2. A program design phase to formulate an intervention through input from an expert panel.
3. A quantitative phase to assess the intervention's effectiveness.

The objectives include understanding the motivations behind GCS, developing an intervention grounded in these insights, and evaluating its impact on women's sexual and Reproductive empowerment. Given the rising prevalence of GCS among unmarried teenagers, this research holds significant importance. It emphasizes the need for comprehensive guidance on GCS for healthcare providers, aligning with empowering women and ensuring access to sexual and reproductive health education.

## Background

Discontentment with one's body and the resulting surge in cosmetic surgeries have evolved into a pressing public health issue in contemporary societies like Iran, notably affecting many girls and young women [1]. In recent years, a growing trend has emerged among healthy women and girls who seek Genital Cosmetic Surgery (GCS) to enhance physical attractiveness, address sexual issues, or both [2]. GCS refers to non-medically necessary cosmetic procedures that alter the structure of the external and internal genitalia in healthy women. These procedures are typically pursued to enhance the aesthetic appearance of the genital area or improve its functionality, with no underlying biomedical concerns or specific medical indications. It's important to note that GCS is distinct from clinical procedures such as gender confirmation surgery, addressing incontinence, treating vaginal prolapse, correcting female circumcision, managing obstetric injuries, accommodating sports-related issues, addressing pain during intercourse, or treating clinically diagnosed female sexual dysfunction. GCS encompasses a broad and expanding array of surgical procedures, including labiaplasty, clitoroplasty, enteroplasty, and hymenoplasty, as well as vaginal rejuvenation, tightening, and reconstruction. These interventions appear to address women's concerns regarding the appearance and functionality of their genital organs [3]. However, specific surgical procedures, such as labiaplasty, may be medically indicated in cases of significant labial asymmetry or hypertrophy resulting from congenital malformations or excessive androgen exposure. Other surgical techniques, such as vaginoplasty of the anterior or posterior compartments and perineoplasty, are typically performed in urogynecology services to address issues like genital prolapse, cystocele, rectocele, stress urinary incontinence, and complications arising from perineal rupture during childbirth [4, 5].

According to statistics published by the Australian government, vulvoplasty or labiaplasty represents the most prevalent form of GCS. In the latest report (2020) from the American Society of Plastic Surgeons, out of the 15.6 million cosmetic procedures conducted, 2.3 million were surgical cosmetic procedures, while 13.2 million were minimally invasive cosmetic procedures. Notably, 92% of all these cosmetic procedures were performed on women. Within this report, 9,725 labiaplasties were documented, indicating the number of operations approved by the association's members. Following nose reshaping, eyelid surgery, facelift, liposuction, and breast augmentation rank as the top five most prevalent cosmetic surgeries. According to the 2020 statistics from the American National Cosmetic Plastic Surgery Database, despite the restrictions imposed by the COVID-19 pandemic, a total of 13,697 labiaplasties were performed [6]. This number indicates an increase compared to 2015 and 2019, which saw 12,903 and 9,945 procedures, respectively.

Interestingly, in 2019, no labiaplasty procedures were recorded for women under 17, possibly reflecting a change in practice following the publication of relevant guidelines and ethical opinions by various professional bodies [7]. Worldwide, the number of labiaplasties performed reached 164,667 in 2019, marking a 24.1% increase compared to 2018 and a substantial 73.3% increase compared to 2015. These figures align with statistics from the American National Institute of Plastic Surgery. However, it's essential to note that reporting cosmetic procedures in the private sector is not mandatory. As a result, this statistic is estimated to be significantly lower than the actual number. This lack of mandatory reporting has contributed to a lack of precise figures regarding the number of these surgeries in Iran [1].

Regrettably, the effectiveness of none of these procedures has been conclusively proven, and the potential for harm must always be considered [2]. Some of these procedures involve the partial removal of vaginal mucosa and the alteration of healthy external genitalia. For this reason, some researchers have contended that there is limited anatomical distinction between GCS and female circumcision [8].

According to the 2016 definition by the World Health Organization, FGM (Female Genital Mutilation) is described as a procedure that involves the partial or complete removal of the external female genital organs or other injuries to the female genital organs for non-medical reasons [9]. Similarly, in 2013, the Royal College of Gynecology and Obstetrics defined GCS as 'non-medical cosmetic surgical procedures aimed at altering the structure and appearance of healthy external or internal female genitalia' [6]. According to these definitions, both practices entail the cutting or removing of female genitalia for non-medical purposes. In this regard, both approaches align with the United Nations' medical definition of injury. FGM is categorized as a harmful practice within the framework of the Sustainable Development Goals (SDGs), as cutting for non-medical purposes is considered a violation of human rights. Since the early 1990s, FGM has been increasingly framed within the context of human rights, extending beyond the realm of limited medical considerations [7, 10]. This redefinition stems from United Nations organizations that condemn the medicalization of female circumcision, as stated in the inter-agency statement, which emphasizes that there is no evidence that the medicalization of female circumcision has reduced obstetric complications or mitigated other long-term complications associated with FGM. Notably, a medical procedure, GCS, carries health risks similar to FGM [11]. Despite various interpretations, in many developing countries where female circumcision is prevalent due to traditional or religious motives, this practice has been medicalized and is conducted by gynecologists or midwives [12].

Power and knowledge are closely intertwined. According to the French philosopher Michel Foucault, power derives from knowledge and is exercised through it. Simultaneously, power reinforces and shapes knowledge. It (re)creates the contexts of its application through knowledge. From this perspective, women and girls can be empowered to their fullest potential through education and knowledge enhancement [13]. Maybe the best time and place for such education and training is integrated into comprehensive sexual education programs for adolescents [14, 15].

Empowering women and girls through education and knowledge is a crucial goal integrated into the SDGs 2030 agenda:

1. SDG 3: Ensuring healthy lives and promoting well-being for all ages, particularly women and girls, by providing universal access to sexual and reproductive healthcare services, education, and information (knowledge).
2. SDG 4: Ensuring inclusive and equitable quality education and promoting lifelong learning opportunities for all. Lifelong learning for adults widens their choices for productive and fulfilling lives, encompassing essential aspects such as gender equality, women's rights, policies to combat gender discrimination, and preventing violence against women and girls.
3. SDG 5: Achieving gender equality and empowering all women and girls while eliminating harmful practices like GCS, considered a form of violence against women [16].

**Fig. 1:**
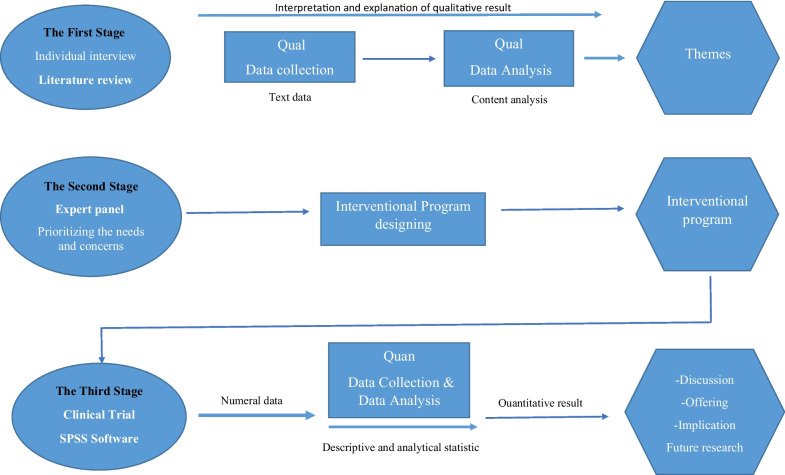
Study visual diagram

In light of the continually evolving shifts in gender norms and the increasing medicalization of sexuality and beauty as societal constructs, it becomes imperative to establish empowerment and awareness programs tailored for women and girls. According to the SDGs, education in sexual and reproductive health, the assurance of women's well-being, and the promotion of women's empowerment are recognized as fundamental rights for women and girls [16]. The primary aim of this study is to formulate and assess an educational initiative to empower women contemplating GCS. This endeavor seeks to enhance the sexual and reproductive health of women and their offspring, enabling them to make well-informed decisions based on their capabilities in this domain (Fig. 1).

In this context, our study employed a multistage sequential approach to develop and assess an intervention tailored to address the specific needs and concerns surrounding cosmetic surgeries of the female reproductive system in Tabriz and Tehran. This intervention was intricately designed to cater to the identified needs and encompass the sexual, psychological, and reproductive concerns of the target population. It aims to enhance women's overall quality of life, focusing on their physical, sexual, and psychological well-being. These goals are not achieved except through the provision of comprehensive documentation and the enrichment of knowledge in this domain, all rooted in the expressed needs and concerns of the target population.

## Objectives

The objectives of this study will be structured into three phases, incorporating both quantitative and qualitative components:

### Objectives of the first phase: qualitative study

1. To elucidate the needs and concerns associated with GCS among women actively seeking these procedures.
2. To gain insights into the needs and concerns of women worldwide considering GCS by conducting a literature review in parallel with the qualitative phase.

### Objectives of the second phase: program design

1. To formulate an intervention grounded in the primary priorities identified during the qualitative stage and informed by the literature review.
2. To prioritize the needs and concerns of women seeking GCS and to validate and endorse the intervention through input from an expert panel.

### Objectives of the third phase: quantitative study

To assess and determine the effectiveness of the intervention designed to address the needs and concerns of women applying for GCS procedures.

## Methods/design

This study employs a multistage combined design conducted in a sequential exploratory fashion. Qualitative data will precede the quantitative phase. We will conduct qualitative research in the initial stage using a contractual content analysis approach. Our goal is to elucidate the needs and concerns of three distinct groups: women seeking GCS, women who have already undergone such procedures, and the partners of these women. Furthermore, we will conduct a literature review during this stage to investigate the global needs and concerns of women considering GCS. In the second stage, we will consider the primary priorities identified in the preceding step to develop the intervention. We will review relevant texts to extract the necessary interventions, plans, recommendations, and strategies to address the needs and concerns of women seeking GCS in Iran and other countries. The final intervention will be chosen through a nominal group meeting attended by experts actively engaged in sexual and reproductive health. Before convening the expert panel for the target group, we will outline the goals and initial content of the interventions with guidance from esteemed professors and advisors. Subsequently, the initial format of the designed interventions will be provided to the experts for evaluation, concurrent with the prioritization of needs and concerns during the expert panel meeting. The selected, qualified intervention will then be implemented in the quantitative phase. The third stage will involve a quantitative study to evaluate and assess the effectiveness of the intervention designed to address the needs and concerns of women seeking genital cosmetic surgeries. We will compile the objectives of the quantitative section based on the identified content and the type of intervention.

### Phase I: qualitative study

We will conduct an initial qualitative study using a contractual content analysis approach during this stage. The primary goal is to elucidate the needs and concerns of women considering cosmetic surgery of the genital tract, including those who have already undergone the procedure, as well as the partners of these women. Additionally, as part of this research phase, we will conduct a comprehensive literature review to investigate the needs and concerns of women seeking GCS globally.

#### Participants

To evaluate the needs and concerns of women in this context, we will conduct semi-structured, in-depth individual interviews. These interviews will involve married women between 18 and 49 who have either sought GCS or undergone such procedures. We will also include their spouses willing to participate in the interview sessions.

#### Inclusion criteria

The inclusion criteria for this study encompass women actively seeking GCS, women with a history of GCS, spouses of female applicants, individuals who are married, fall within the reproductive age range (18–49 years), do not have a diagnosed physical or mental illness, are literate in reading and writing, and are proficient in speaking both Persian and Turkish languages.

#### Sampling

In the qualitative study, the research population will be drawn from individuals with experience or knowledge relevant to the topic under investigation. Specifically, in this qualitative phase of the study, we will target women attending gynecological clinics at Tabriz University of Medical Sciences and Tehran University of Medical Sciences who possess basic literacy skills in reading and writing. Our sampling approach aims to attain maximum diversity among participants. Selection will be based on various factors, including age, education level, socio-economic status, number of pregnancies and births, and marital status (widowed, single, and married). Sampling will continue until a sufficient amount of data is collected.

#### Research environment

Interviews will be arranged at the convenience of the participants and conducted in locations where they feel comfortable and trust the interviewer. These settings may include hospitals, health complexes associated with the University of Medical Sciences, clinics, universities, participants' homes, and similar locations.

#### Data collection process

Data collection will commence upon obtaining approval for the research plan and receiving ethical clearance from the Joint Ethics Committee of the Faculty of Nursing and Midwifery and the Faculty of Rehabilitation. Additionally, a written letter of introduction will be obtained from both Tehran University of Medical Sciences and Tabriz University of Medical Sciences.

The initial data collection phase will involve in-depth interviews with women with a GCS history and willing participants' spouses. These interviews will follow an interview guide. The number of interview sessions will be determined based on individual participant conditions and responses to the research questions. Before the interview, the researcher will ask the participants to review and sign the informed consent form. Interviews will be scheduled at a time and location preferred by the participants, ensuring their comfort and upholding principles of privacy and confidentiality.

Establishing effective communication with the participants is crucial at the interview's outset. This approach can be achieved by providing a brief, understandable explanation of the significance of the research, which helps build trust before delving into the interview. All interview transcripts will undergo anonymization, and the interview recordings will be identified with numerical codes to safeguard privacy. Participants will receive assurance that their information will remain confidential and that their names will not be disclosed. Subsequently, participants will be invited to discuss their needs and concerns related to their genital area. Follow-up questions will be tailored based on initial responses and the interview guide. If necessary, probing questions like "What do you mean?" or "Could you elaborate further?" may be used during the interview. As the study progresses, interview questions may be adjusted or new ones added based on emerging themes and researcher inquiries. Indeed, here are the interview questions:

1. What prompted your interest in pursuing GCS, and what specific goals are you aiming to achieve through this procedure?
2. Could you share the primary motivation behind your decision to undergo GCS?
3. Did any external influences, such as your spouse, family, friends, media exposure, or medical professionals, play a role in your decision-making process?
4. From your perspective, why do you believe some women opt for cosmetic surgery on their genitals?
5. What are your expectations regarding the outcomes of this surgery, particularly concerning its potential effects on reproductive health and your sexual well-being?
6. Could you elaborate on your expectations and what you hope to achieve through this surgery?
7. Regarding the concerns that led you to consider this surgery, do you believe they can be assessed before the procedure, or do you anticipate addressing them afterward?
8. Would you like to discuss or express any specific worries or considerations related to the surgery?

Once the interview questions have been addressed, participants can share any additional thoughts or concerns. Towards the interview's conclusion, we will explore the potential for further discussions if necessary. All interviews will be recorded and transcribed verbatim, with the participant's permission. We prioritize confidentiality, and as a measure to ensure accuracy, we will provide the interview text to the participant for their review and confirmation.

#### Data analysis

In the qualitative phase of this study, data analysis will be conducted using the content analysis approach facilitated by MAXQDA software version 12. Our approach is conventional content analysis, an inductive method for analyzing textual data. This method is one among several research techniques used for text analysis.

The recorded interviews will be transcribed verbatim, and the text will undergo thorough and repeated analysis to ensure the researcher's deep immersion in the data. Analysis units will be identified, classified, and coded. Determining the analysis units is critical in content analysis, as they constitute the primary content within the texts under examination. The research team, including the supervisor and advisor, will review and validate the data classification and coding. Codes will be organized into classes and subclasses and assigned to their respective categories based on semantic and content similarities after undergoing multiple rounds of review.

In the subsequent stage, code stability within the text will be validated through testing and obtaining consensus among most research team members. Two external experts with experience will review the codes assigned to the categories and subcategories to ensure coding stability. The following stage will conclude the accurate categorization of data and the given codes. The characteristics and relationships within and between categories will be examined and analyzed. Each category and subcategory will be explored alongside the interview text, and ultimately, the formed categories will be interpreted and reported.

#### Accuracy and reliability of qualitative data

In the current study, various criteria, such as validity, reliability, verifiability, transferability, and accuracy, will be employed to ensure the accuracy of qualitative data.

The researcher will actively collect and analyze data for an extended period to enhance validity. They will establish a strong rapport with participants by thoroughly reviewing and critically examining the interviews. Additionally, the research team will incorporate data revised by participants and team members, ensuring maximum diversity in socio-economic status. These measures will contribute to the research's validity.

To achieve and enhance reliability, the research team and external observers will develop, pilot, and refine the interview guide, coding, and analysis processes multiple times. This iterative approach aims to comprehensively address women's needs and concerns in the reproductive health field.

To ensure verifiability, which entails reflecting the participants' voices rather than the researchers' biases, the researcher will seek input from two external experts knowledgeable about qualitative research methods. These experts will provide feedback on the text coding process and interpretations made during several interviews.

Lastly, to ensure transferability, which refers to the generalizability of the study, efforts will be made to collect information from participants with diverse characteristics, including literacy, age, and socio-economic status. This approach will enhance the study's ability to apply findings to a broader context.

### Phase II: designing interventional program

During the study's second phase, we will undertake a two-step process to formulate an intervention that addresses the needs and apprehensions of women considering cosmetic surgery for their genital area. These steps involve:

#### Textual analysis and review

This stage is the primary prioritization of concerns and the development of appropriate interventions**.** In the second phase, we will assess interventions and programs related to the needs and concerns associated with GCS, both in Iran and other countries. To ensure a comprehensive search, we have utilized various databases, such as SID, Magiran, Iran Medex, ProQuest, PubMed, Web of Science, Scopus, Sage, and Embase. Our investigation encompasses the period from the year 2000 up to September 2023. We have conducted database searches using diverse combinations of keywords in both Persian and their corresponding Latin equivalents. These keywords encompass "genital cosmetic surgery," "sexual health," "women," "labiaplasty," "genital self-image," "qualitative research," "mixed method," "female," "anesthetic surgery," and "genital self-image."

#### Conducting an expert panel discussion

Based on the first-stage research findings, the panel of experts will prioritize identified needs and concerns. The development of the intervention will heavily rely on the opinions of these experts, with a focus on the most crucial interventions and programs. Before convening the expert panel meeting for the target group, the research team, guided by esteemed professors and advisors, will define the goals and initial content of the interventions addressing these needs and concerns. Subsequently, the preliminary format of the designed interventions will be presented to the experts during the panel meeting. During this meeting, experts will prioritize the identified needs and concerns concurrently. The highest quality intervention will then be implemented in the quantitative phase. An expert meeting will be organized and conducted as a nominal group, and small group discussions (Focus Groups) will be structured to address this. This meeting will involve experienced experts with a history of providing services in sexual and reproductive health, specifically women's health. The participants will include specialists in reproductive health, psychiatrists, gynecologists, midwives, nurses, and psychologists.

Utilizing the Focus Group approach is essential, as it facilitates the extraction of diverse ideas and opinions and encourages collective thinking. This approach is precious in shaping the intervention to address concerns related to women seeking cosmetic surgeries for the genital system.

The Template for Intervention Description and Replication (TIDieR) tool will assess the designed intervention [17]. Given the need for a comprehensive intervention description to make informed selections, we will utilize the TIDieR checklist and model guide to detail and replicate the intervention. The TIDieR checklist comprises 12 essential items:

1. Brief name: provides a succinct name or phrase describing the intervention.
2. Why: explains the underlying logic, theory, or purpose of essential intervention components.
3. What (materials): describes any physical or informational materials used in the intervention, including those provided to participants or used in its delivery.
4. What (procedure): details the location, strategies, activities, and processes involved, including any supportive activities.
5. Who provided: specifies the expertise, background, and specific training of intervention providers.
6. How: describe the delivery methods (e.g., face-to-face or online) and whether they are individual- or group-based.
7. Where: identifies the locations where the intervention occurs, including necessary infrastructure.
8. How much: addresses the intervention's quantity or dose.
9. When: specifies the frequency of the intervention.
10. Tailoring: focuses on customizing intervention details, such as session duration, frequency, and intensity.
11. Modification: describes any adaptations or changes made during the study, including reasons, timing, and implementation.
12. How well (planned): discuss methods for assessing adherence to the intervention and strategies for maintaining or improving compliance.

The checklist enhances the reporting process and repeatability of interventions, ensuring that the most suitable intervention is selected through quantitative evaluation by a group of experts. Before this research's quantitative stage, 8 to 10 field experts will complete and review the checklist to assess the intervention thoroughly.

### Phase III: quantitative study

#### Type and approach

In the quantitative phase of this mixed-method research, a semi-experimental trial will be conducted, comprising both intervention and control groups. This phase, the second stage of the study, will primarily concentrate on the quantitative aspect, specifically the intervention test.

The third stage of this research involves a quantitative clinical trial. Its objective is to evaluate the effectiveness of the intervention designed to address the needs and concerns of women seeking cosmetic surgery for the genital system.

#### Population

In this study stage, the participants will be chosen from married women aged 18–49. These women will attend women's clinics at hospitals affiliated with Tabriz University of Medical Sciences and Tehran University of Medical Sciences.

#### Inclusion criteria

To participate in the trial phase of the study, individuals must meet the following criteria:

1. Absence of physical or mental illnesses or the use of specific medications
2. Possess minimal literacy
3. Express a desire to take part in the study
4. Not be pregnant
5. Not be breastfeeding

#### Exclusion criteria

Participants will be excluded from the quantitative phase of the study if they meet any of the following criteria:

1. Congenital disabilities of the genital system.
2. History of burns or cancer in the genital area.
3. Various degrees of female circumcision.
4. Confirmed sexual dysfunction (FSFI ≤ 26).
5. Multiple degrees of pelvic organ prolapse.
6. Non-cooperation of participants in continuing to attend training sessions.
7. Occurrence of pregnancy during the course.

#### Research sample

In this research stage, the sample will comprise married women who visit women's clinics at hospitals affiliated with Tabriz and Tehran Universities of Medical Sciences. These women have expressed concerns and anxiety about their genital area and are seeking cosmetic surgery for this specific concern.

#### Research environment

The research will be conducted in women's clinics within educational hospitals affiliated with Tabriz and Tehran Universities of Medical Sciences. In cases where access to eligible participants in government centers may be challenging, sampling from private sectors will be explored because most of these surgeries are performed in private centers. Additionally, posters will be displayed at epilation and laser hair removal centers to maximize participant recruitment, inviting individuals to participate in the study.

#### Sample size

The sample size for this study was determined using G-Power software, considering various aspects of genital self-image. Referring to the research conducted by Weitkamp et al. [18], with values M1 = 29.09, M2 = 24.11 (assuming a 20% increase due to the intervention's impact), SD1 = SD2 = 3.22, a two-sided α of 0.05, and a Power of 90%, the sample size for each group was initially computed as 15. The final sample size for each group was established at 17 individuals to accommodate a potential 10% dropout rate.

#### Sampling method and randomization

The researcher will initiate the process by obtaining ethical approvals from the Tehran University of Medical Sciences. Following this, they will secure the required legal documents and permits from the Medical Vice-Chancellor of Tabriz and Tehran Universities of Medical Sciences. If necessary, this will allow them to conduct sampling within hospitals, clinics, and facilities under the university's jurisdiction, including private properties.

Subsequently, the researcher will visit the gynecological clinics within these facilities. In collaboration with the department head and resident urogynecology specialist, they will identify individuals seeking GCS within this clinical setting.

The initial sampling will employ a convenience sampling method, with individuals expressing an interest in participating in the study. They were subsequently screened against the eligibility criteria. Eligible participants will then be categorized into two groups: the intervention group, which will receive education tailored to address genital area needs and concerns, and the control group, which will receive sexual health and reproductive education.

An independent individual not involved in the research will generate an allocation sequence using a computer program (randomizer) to allocate participants. The allocation process will be concealed by writing the type of training assigned to each participant on paper, which will then be sealed inside consecutively numbered opaque envelopes.

Following the entry of each eligible participant into the Study (after obtaining informed consent and collecting primary data), the envelopes will be opened sequentially, and the participant will be assigned to a group according to the order of entry into the study.

#### Participant recruitment and data collection

Upon identifying eligible participants, informed consent will be obtained, and the participants will proceed to complete the study questionnaires. Given that many genital cosmetic surgeries are conducted in hospitals and private clinics, the researcher will initiate sampling in these facilities after making the necessary arrangements in case access to potential participants in these settings is limited.

Following the intervention sessions in both the intervention and control groups, changes in the participants' awareness and attitudes regarding the decision to undergo cosmetic surgeries will be reassessed.

Regarding blinding, this study will employ one-way blinding. Due to the nature of the intervention, it is not feasible to blind the researcher or the participant. However, to ensure the blinding of the outcome assessor, the questionnaires will be administered and completed by the researcher's assistant.

#### Study variables and outcomes

After the trial phase, the following variables and outcomes will be examined:

Independent Variables (Educational Interventions): These are the interventions designed and implemented as part of the study.

Dependent Variable: The primary dependent variable is the decision to undergo or refrain from GCS, which will be assessed at the end of the trial phase.

##### Primary outcomes

Intention to undergo or actual performance of genital cosmetic surgeries.

##### Secondary outcomes.

1. Women's Female Sexual Function Index (FSFI) before and after the intervention.
2. Women's Female Genital Self-Image Scale (FGSIS) before and after the intervention

#### Data collection process

Attitudes toward GCS: Participants' attitudes and opinions regarding GCS will be assessed using items developed during the qualitative phase of the study. These items will provide insights into participant's perceptions and concerns regarding these procedures.

In the quantitative phase of this study, we will employ a researcher-developed questionnaire, which has been meticulously designed and validated based on the findings from the qualitative phase. After securing informed consent, we will initiate data collection using this questionnaire. The initial data will encompass participants' demographic, sexual, and reproductive characteristics. Additionally, participants will be asked to complete the standard FSFI [19] to assess sexual function and genital self-image to evaluate by using the FGSIS [20].

1. The FSFI is comprised of 19 items categorized into six sub-domains, each assigned a specific weight or coefficient. These sub-domains include sexual desire (2 items with a coefficient of 0.6), sexual arousal and orgasm (4 items with a coefficient of 0.3 each), orgasm and satisfaction (3 items with a coefficient of 0.4 each), and pain (2 items with a coefficient of 4/0). Participants will respond on a Likert scale ranging from 0 to 5. A score of zero signifies no sexual activity within the past 4 weeks. After applying the coefficients, the overall score will fall between 2 and 36, with a lower score (FSFI ≤ 26) indicating less favorable sexual performance [21].
2. FGSI is a 7-item patient-reported assessment tool designed to evaluate one's genital self-image. Participants rate each FGSIS item using a 4-point Likert-type scale, with options ranging from 1 (strongly disagree) to 4 (strongly agree). The individual item scores are summed to calculate the total score, falling within a range of 7–28 points. Higher total scores on the FGSIS signify a more positive genital self-image [22].
3. The demographic profile questionnaire will encompass inquiries related to socio-economic status, including age, marital status, educational background, occupation, income, number of pregnancies and deliveries, and any history of illness or medication usage.

Furthermore, data collection will include an assessment of participants' attitudes toward undergoing GCS. The questions for this assessment were formulated during the qualitative phase of the study.

#### Analysis approach

Analyzing the gathered data is vital in deriving valuable insights from it. We will employ descriptive statistics to analyze the collected data, including the mean, standard deviation, minimum, and maximum measures. Additionally, we will utilize inferential statistics methods such as independent t-tests, analysis of covariance, paired t-tests, chi-square tests, Fisher's exact test, and Mann–Whitney tests. This comprehensive analysis will be conducted using SPSS software.

## Discussion

Over the past 150 years, there has been a notable rise in medical oversight and scrutiny of sexual behavior and lifestyles. This surge has brought various medical interventions, including diagnostics, psychotherapy, psychiatry, surgery, and pharmaceuticals. Furthermore, many healthcare professionals have become engaged in addressing multiple sexual matters. Simultaneously, sexual health has garnered substantial attention from the media and various organizations. This phenomenon, known as the medicalization of sex, has evolved into a multifaceted domain that intersects with gender, sexuality, professional education, medicine, technology, rapid social transformations, global capitalism, culture, and politics [23, 24].

Medicalization is a dynamic concept that highlights the growing impact of medical ideologies, institutions, and individual figures. Typically, this term signifies a phenomenon in which issues not inherently medical are redefined and addressed as medical problems, often framed as diseases or disorders. It can also denote a trend in which medicine progressively extends its influence into various facets of daily life [25].

The rise of procedures like vaginal rejuvenation and labiaplasty in GCS represents a form of pharmaceuticalization within the broader context of the medicalization of sexuality. This development has sparked debates and garnered significant interest. Critics of the medicalization of sex typically raise concerns about the overemphasis on genital aspects of sexuality, the imposition of standardized sexual norms (which can lead to increased feelings of shame), the neglect of psycho-social factors that influence sexual well-being, identity, practices, and standards, as well as the potential health risks and unintended side effects associated with both approved and off-label medical treatments [26].

Feminist medical literature challenges the male-dominated field of medicine, which historically centered on androcentric (male-focused) perspectives primarily oriented around disease and illness. These works shed light on the marginalized position of women within the field, highlighting the historical exclusion of women from early medical professionalization and the persistent negative attitudes toward women's bodies in medical discourse and education. Women's life experiences, including menstruation, childbirth, menopause, and cosmetic surgery (especially in the genital region), are significantly influenced by medical practices. Additionally, women often find themselves more exposed to medicalization due to their roles in overseeing family healthcare [24, 27].

The term medicalization often underscores the adverse consequences, such as overtreatment, overdiagnosis, and the oversight of non-medical aspects of life problems, which some may refer to as overmedicalization. Nevertheless, many have explored the positive and negative dimensions of this phenomenon. They identify individuals who may be categorized within the medical framework instead of stigmatizing them for perceived mental health issues [23, 28].

The vulva, a part of the female body, has often been inaccurately described and misunderstood, partly due to historical and social constructs. Notably, dictionary definitions of male genital anatomy emphasize function, while those for female genital anatomy mainly mention position [29]. What defines a normal female reproductive system is an understudied area in medicine [30]. Few articles discuss the measurement of female genital organs, and the criteria for hypertrophy and normality differ. There's no standard for measuring and describing natural female genital anatomy, and medical textbooks lack details about the range of variation and measurement [31]. GCS aims to enhance the appearance of the female genitalia, explicitly addressing labial hypertrophy. However, there's limited evidence defining the spectrum of labial hypertrophy and what constitutes the average size, color, and shape of labia [32].

The importance of developing educational and counseling interventions cannot be overstated, especially when contrasted with clinical procedures like cosmetic surgery, which is invasive and lacks medical justification. Midwives are often the initial point of contact within the gynecological and midwifery care system, making them pivotal in educating girls and women about the diverse appearances of the genital system and the associated risks of genital surgery. Therefore, well-informed midwives can significantly enhance women's health by addressing modifiable psycho-social factors [33]. In this context, doctors, midwives, and reproductive health specialists must possess the knowledge to empower women and girls in this domain [34].

## Conclusion

This study marks the first attempt to design and assess an intervention addressing the needs and concerns of cosmetic surgeries performed on the female genital and reproductive system. The hope is that this study's compilation and implementation will yield substantial evidence and documentation regarding the impact of educational interventions on women's and girls' sexual and reproductive empowerment. Given the rising prevalence of GCS, even among unmarried teenagers, this approach is of utmost significance. It underscores the necessity for gynecological and midwifery service providers to have comprehensive guidance on GCS. Such guidance can be an essential resource for healthcare providers in this field.

## Data Availability

Not applicable.
